# Cases from the Diary of a Physician

**Published:** 1836

**Authors:** 


### WESTERN JOURNAL

OF THE

### MEDICAL AND PHYSICAL SCIENCES:

**For July, August, and September, 1836.**

### ESSAYS AND CASES.

### Art. I.—Cases from the Diary of an old Physician.

*No.* 1. *Nervous Irritability.*—Mrs. H. B. aged 24 years, of a very nervous temperament, while sewing on the 15th September, 1824, pricked her left thumb under the nail, with a small cambrick needle. Little attention was paid to so trifling a hurt until the 17th inst., when it became tender and painful, but without any signs of inflammation. The pain then began to ascend the arm, and was so severe as to become alarming. I was called on for advice, and directed the part to be bathed with hot spts. tereb. and laudanum, and to take inwardly laud. and vitr. æther. sufficient to allay the pain. By night it had increased, and begun to affect the muscles of the face and the neck on the wounded side to such a degree as to raise serious fear of a trismus or "lock jaw." The doses of laud. and æther were increased to the extent of 120 drops, every hour or two, and a linament of ol. succin. ol. oliv. and laud. rubbed hot on the arm and neck, with a direction to keep the arm very warm with a covering of flannel, as cold air seemed to increase the pain very much. On the 18th a blistering plaister was applied all round the thumb, with the intention of relieving the irritation of the wounded nerve, by exciting a new action in the neighboring parts. This application, aided by the warm bath, had a very beneficial effect in allaying the pain and spasmodic tendency of the disease. By continuing the laudanum in large doses, sometimes to the extent of half an ounce, the pain was in the course of a week so far subdued as only to be troublesome occasionally. The disease now put on more the character of hysteria, attended at times with violent delirium, and fits of sobbing. For this difficulty, venesection was prescribed with manifest advantage. She was also put upon a course of carb. iron, with the intention of strengthening the nervous system, as well as the general health, which had be come considerably impaired. By the first of November, the pain had nearly ceased and the symptoms of trismus left her; but the whole arm had become so irritable that the least cold was painful. To overcome this sickly irritability, the cold bath, or rather shower bath, was directed every morning, and even at night, if necessary, with decided benefit, the arm remaining comfortable and easy for hours after the application of the water. About the middle of the month of November, electricity was tried, by passing light shocks through the hand and arm, without much apparent benefit. Near this period she became pregnant, which probably occasioned several severe fits of hysteria. A painful affection, with inflammation, of the face and jaws came on in December, which suppurated and discharged from the gum of the affected side. This, with the change in the system, consequent on pregnancy, relieved the train of spasmodic symptoms which had so long attended her. From this time she continued in tolerable health until June, when she was troubled with a good deal of pain in the left side, attended with nervous symptoms; bleeding, with nervines relieved these difficulties. About the middle of July she was delivered of a dead child. The fatigue of the labor, and the depression of mind, from the loss of the child, brought on a hoarseness and soreness of the throat, attended with severe pain in the chest, great difficulty of breathing, and a cough which sounded like the barking of a small dog. There was no expectation. This was the second night after the "accouchment." To relieve these symptoms, two or three free bleedings were directed; and as the blood was highly inflammatory, the evacuations were large, averaging twenty ounces each. A large blistering plaister was applied to the breast, febrifuge and expectorating powders and drinks were given, with a diet of gruel and rice water. This course afforded relief in two or three days. It also checked the secretion of milk so much, that a small puppy kept her breasts free and soft. After these symptoms had left her, there commenced a very severe pain in the back part of her head; it was apparently occasioned by nervous irritation, but so severe that it required the largest doses of opium to afford any relief. These were repeated so regularly, and so often, that the worst consequences were feared from their continued use. Thinking it might arise from debility, as the paroxysms were periodical, large doses of sulp. quinine were given a few hours previous to the accession of the pain. This medicine had a fine effect for a few days, in preventing the return of the paroxysms; but in a week or more it ceased: it seemed to loose its influence over the complaint. Riding in a carriage was now tried with the happiest effect, the pain left her after riding a few miles, and by continuing it a number of days, and changing her place of residence as long, the pain in the occipital region left her, and did not again return. Through August and September, her health improved, but at the setting in of cool weather the last of October and beginning of November, the pain returned, but was now seated in the sternum, apparently behind the bone or in the adjoining mediastinum. It was attended with little or no fever, but with considerable rigors—was very acute and felt like the gnawing of some small animal. A large blister relieved it after a few days. In a week or two, a similar pain was felt in the right side, about the seventh rib, midway between the scrobiculus cordis and spine. It was very severe, and attended with so much fullness of the side, that she was fearful that an abscess was forming. It was probably occasioned by venous engorgement of the liver. I did not see her in this attack for several days after its commencement, but a neighboring physician saw her; bled and blistered her, calling it a pleurisy. The blood which I saw was not sizy, nor did it present an inflammatory appearance; tongue not coated, nor was there heat or thirst. I therefore concluded the pain arose from the same cause as that formerly in the head and breast, viz. nervous irritation; perhaps the remains of that action excited in the system from the prick of the needle, and now wandering through the frame like a rheumatic affection. With this view, refraining from any more bleeding, I prescribed an infusion of R. Vallerian and Fol. Eup. perfol. warm, so as to produce perspiration, with occasional doses of Tolu. bals. comp. and after a few days put a seton in the side, over the seat of the pain, with a direction to make it discharge freely, and as a tonic, to take hoarhound and the mur. tinct. fer. The seton was applied to relieve engorgement, and prevent the fixing of disease on a vital part, by the long continuance of pain; it had also been used with benefit in her neck, the last summer, while laboring with the distress in her head. The pain left her as soon as the seton began to discharge, and by the use of the tonic medicines her health was much improved; the pain occasionally returns, before changes of weather, like chronic rheumatism. Remarks—In the above case we learn how trifling an injury may derange the healthy movements of the human frame, and how the derangement is increased ten fold by nervous temperament. The more sensititive the mind and intellectual faculties, with so much less impunity the subject bears physical injury. A simple cut or bruise on an ignorant robust man, heals much more readily than on one with a refined mind and delicate frame. The animal man bears injury much better than the intellectual man. H.

*Marietta*, 27 *April*, 1836.

